# Efficacy and safety of avacopan in the treatment of ANCA-associated vasculitis: a systematic review and meta-analysis

**DOI:** 10.1186/s41927-025-00569-w

**Published:** 2025-10-03

**Authors:** Khaled Aldhuaina, Khawla Alghanim

**Affiliations:** Rheumatology Unit, Department of Internal Medicine, King Fahad Military Medical Complex, Dhahran, Saudi Arabia

**Keywords:** Avacopan, ANCA-associated vasculitis, Systematic review, Meta-analysis, Glucocorticoid-sparing, Vasculitis treatment

## Abstract

**Background:**

Antineutrophil cytoplasmic antibody (ANCA)-associated vasculitis (AAV) is a dangerous autoimmune condition that usually requires high-dose glucocorticoids with immunosuppressive agents. Although effective, long-term glucocorticoid use is associated with significant toxicity. Avacopan, a selective inhibitor of C5a receptors, has emerged as a possible glucocorticoid-sparing drug for AAV, potentially offering a safer, more specific approach to treat the disease. This systematic review and meta-analysis aimed to compare the efficacy and safety of avacopan and conventional glucocorticoid-containing regimens for the treatment of AAV.

**Methods:**

A systematic search was conducted in PubMed, Embase, Scopus, and the Cochrane Library between 2000 and 2025. Post hoc subgroup analyses, randomized controlled trials, and observational reports that compared avacopan with glucocorticoid regimens in GPA or MPA patients were included. The primary outcomes were remission at week 26 and sustained remission at week 52. The secondary outcomes were relapse rates, renal outcomes, adverse events, glucocorticoid toxicity, and health-related quality of life.

**Results:**

Nine studies with 2080 patients were combined. Compared with glucocorticoids, avacopan was associated with noninferior remission rates at week 26 and significantly higher sustained remission rates at week 52 (RR = 1.02, 95% CI: 0.88–1.19). It was associated with significantly reduced glucocorticoid toxicity, fewer adverse events, and improved quality of life scores. Heterogeneity was low (I^2^ = 5.4%), which supported the consistency of the results.

**Conclusions:**

Compared with standard glucocorticoid treatment, avacopan appears to offer a safer alternative with similar disease control and significantly reduced toxicity. These findings suggest a potential shift towards less toxic and more personalized approaches in AAV management, though further research is warranted to confirm these benefits.

**Trial registration:**

PROSPERO CRD420251033866.

**Clinical trial number:**

Not applicable.

## Background

Antineutrophil cytoplasmic antibody (ANCA)-associated vasculitis (AAV) is a group of severe, autoimmune, multisystem diseases characterized by the inflammation and necrosis of small- and medium-sized vessels [1]. The principal clinical subtypes of AAV are granulomatosis with polyangiitis (GPA), microscopic polyangiitis (MPA), and eosinophilic granulomatosis with polyangiitis (EGPA) [2]. These conditions are characteristically associated with the presence of circulating ANCAs against PR3 or MPO antigens. AAV particularly targets the kidneys and respiratory tract but can involve virtually any organ system, leading to diverse clinical manifestations, such as renal insufficiency, pulmonary haemorrhage, sinusitis, and peripheral neuropathy [3]. AAV pathogenesis is modulated by the interaction of ANCA autoantibodies with neutrophils and engagement of the alternative complement pathway, most critically via the C5a receptor. This process results in a cascade of inflammation perpetuating endothelial injury, vascular inflammation, and resulting tissue damage. The clinical course of AAV ranges from a local mild disease to potentially fatal multisystem disease. The prognosis has been far more favourable in recent decades because of the introduction of immunosuppressive therapy, but relapses and complications related to treatment are still major issues [4, 5].

AAV treatment generally consists of two phases: induction of remission and remission maintenance. Induction therapy has generally been the cornerstone, with high-dose glucocorticoids in combination with cyclophosphamide or rituximab [6, 7]. Cyclophosphamide, a member of the alkylating class of drugs, is effective but is associated with the potential for major side effects, including cytopenias, infections, and a chronic risk of malignancy. Rituximab, which is a CD20-positive B-cell monoclonal antibody, has been studied as an alternate induction agent and has been shown to be equally effective as cyclophosphamide at inducing remission [8]. Glucocorticoids, although indispensable in the short-term management of the disease, are beset by a crippling side effect load with high-dose, protracted administration [9]. These side effects include disturbances in metabolism, osteoporosis, cardiovascular problems, and an increased risk of infection. Once induced, maintenance therapy typically involves less severe immunosuppression with agents such as azathioprine, methotrexate, or low-dose rituximab. Despite remission, patients are likely to experience relapse, which may necessitate repeated induction therapy and prolonged maintenance immunosuppression, leading to the cumulative burden of therapy. The most vexing dilemmas in AAV treatment are what strategies to use to balance disease control with the prevention of treatment-related side effects, particularly those related to long-term glucocorticoid administration. While glucocorticoids continue to be the staple of remission induction, strategies that reduce or obviate their use without compromising disease control are being pursued more vigorously [10, 11].

The use of glucocorticoids, while beneficial, has increasingly come under attack because of the profound toxicity of prolonged therapy. Glucocorticoid toxicity has been shown to be a cause of morbidity and mortality in AAV patients, particularly those receiving prolonged therapy for relapsing disease [12, 13]. Thus, the development of glucocorticoid-free regimens that are effective but as benign as possible is urgently needed. Research in the past few years on the pathophysiology of AAV has implicated the involvement of the complement system and, more significantly, the C5a receptor as a drug target. The oral C5a receptor antagonist avacopan has been evaluated as a glucocorticoid-sparing agent in the treatment of AAV, including in both randomized trials and early real-world studies [14]. By inhibiting the activation of C5a receptors, avacopan prevents neutrophil activation and endothelial damage without blocking the complete complement cascade and thus does not set the stage for infection, as observed with other panspectrum immunosuppressive approaches [12, 15–17].

The ADVOCATE trial and more recent trials have indicated that avacopan combined with conventional treatment has the ability to substantially maintain the remission of AAV at reduced doses along with minimized glucocorticoid toxicity. These findings represent a paradigm shift for the treatment of AAV, with an attenuated treatment plan that is better suited [18]. The adverse effects of glucocorticoids will hopefully decrease. Therefore, this systematic review and meta-analysis aimed to assess the safety and efficacy of avacopan compared with conventional glucocorticoid therapy in patients with AAV. The main objective of this study was to assess the clinical efficacy of avacopan in inducing remission and long-term remission and its ability to reduce glucocorticoid toxicity. The secondary endpoints were renal outcomes, quality of life measures, relapse rates, and overall safety profiles, including the rate of adverse events [19–21].

The questions addressed by this study are as follows: How does avacopan compare with standard glucocorticoid regimens in achieving sustained remission of AAV? What is the impact of avacopan on glucocorticoid toxicity in patients with AAV? Do avacopan and standard treatment compare with regard to renal outcomes and quality of life? What are the safety implications of avacopan use, particularly for serious adverse events and infections? Through the synthesis and systematic review of the available evidence, this study aims to provide a comprehensive overview of the use of avacopan in the treatment of AAV, guide clinical practice, and clarify directions for future research.

## Methods

### Search strategy

The search strategy in this systematic review and meta-analysis was to conduct a wide-scale literature search in a variety of databases, such as PubMed, Scopus, the Cochrane Library, and Embase [22]. These databases were selected since they index a vast array of the biomedical literature, including reviews, clinical trials, observational studies, and meta-analyses. The date range for the search was January 2000 to March 2025, thereby capturing the latest studies relevant to the research question. A combination of Medical Subject Headings (MeSH) keywords and terms was used to incorporate sensitivity and specificity in the search. ANCA-associated vasculitis, avacopan, glucocorticoids, rituximab, cyclophosphamide, and treatment outcome were some phrases used in the search strings along with Boolean operators such as AND and OR. Additionally, the reference lists of the available articles were screened manually to identify other studies meeting the inclusion criteria. The protocol for this systematic review and meta-analysis was prospectively registered in the PROSPERO database (registration number: CRD420251033866). Clinical trial number: not applicable.

### Eligibility criteria

The inclusion criteria used to select the studies were delineated according to the PICO model, namely, the population, intervention, comparison, and outcomes (Table 1). Studies were analysed if they included patients with AAC, namely, GPA or MPA, as evidenced by the clinical presentation, serological activity (PR3-ANCA or MPO-ANCA), or histopathology. The comparator of interest was avacopan administered as part of an induction or maintenance therapeutic regimen, with or without other first-line therapies such as rituximab or cyclophosphamide. The comparator was initial glucocorticoid-based regimens with or without the use of adjunct immunosuppressive drugs. The studies were restricted to peer-reviewed clinical trials, observational accounts, and meta-analyses reporting the efficacy, safety, or glucocorticoid-sparing effects of avacopan in patients with ANCA-associated vasculitis. Only English publications were included as eligible studies. Quantitative research reporting outcome data for remission rates, relapse rates, renal function, quality of life, adverse events, or glucocorticoid-induced toxicity were prioritized for inclusion [23].

**Table 1:** Presents the PICO framework that guided study inclusion criteria

Component	Description
Population	Patients diagnosed with AAV (GPA or MPA)
Intervention	Avacopan administered as a monotherapy or in combination with rituximab or cyclophosphamide
Comparison	Conventional glucocorticoid-based regimens, with or without adjunctive immunosuppressive agents
Outcomes	Remission rates (week 26 and 52), sustained remission, relapse rates, renal outcomes, HRQoL, adverse events

### Data extraction

The data were extracted independently by two reviewers using a pre-standardized data extraction form. The extracted variables were study characteristics, such as authors, year of publication, study design, sample size, and follow-up duration. Patient demographics, such as age, sex, disease subtype, and ANCA status, were also recorded. Interventions, such as the dose and duration of avacopan and concurrent therapies, were recorded. Efficacy outcomes, such as week 26 and 52 remission rates, sustained remission rates, relapse rates, and estimated glomerular filtration rate (eGFR) changes, were recorded. Safety outcomes, such as the adverse event incidence, glucocorticoid-related toxicity, and serious treatment-emergent adverse events (TEAEs), were also recorded. Other outcomes assessed included health-related quality of life (HRQoL) measured by validated tools such as the EuroQol-5 Dimension (EQ-5D) or the Vasculitis Damage Index (VDI).

### Risk of bias assessment

The risk of bias assessment was performed with known tools for different study designs. For randomized controlled trials (RCTs), the Cochrane Risk of Bias Tool was applied to quantify potential biases associated with randomization, allocation concealment, blinding, incomplete outcome data, selective reporting, and other biases [24]. For nonrandomized trials, the Risk Of Bias In Nonrandomized Studies of Interventions (ROBINS-I) tool was employed to quantify biases for confounding, participant selection, outcome measurement, and reporting. Independent reviews of every trial were performed by two reviewers, and if discrepancies arose, a discussion or consultation involving a third reviewer was performed. Studies at a high risk of bias were included in the sensitivity analysis to ascertain whether they affected the overall results [25].

### Data synthesis

The synthesis of data was accomplished by a qualitative synthesis of the included studies. A qualitative overview was provided for all of the studies of the efficacy and safety of avacopan versus usual glucocorticoid-based therapies. When quantitative information was available, quantitative data were pooled with the assistance of meta-analysis techniques. The principal outcomes assessed were remission at weeks 26 and 52, sustained remission, and reduced glucocorticoid toxicity. The secondary outcomes assessed were relapse, renal disease, HRQoL, and adverse event occurrence. Statistical calculations were performed using review manger (RevMan) version 5.4.1 (Cochrane Collaboration), and the findings are reported as risk ratios (RRs) for dichotomous variables and mean differences (MDs) or standardized mean differences (SMDs) for continuous variables with 95% confidence intervals (CIs) [26]. Heterogeneity was calculated using the I^2^ statistic, and 25, 50, and 75% were used as the points for low, moderate, and high heterogeneity, respectively [27]. If significant heterogeneity existed, a random effects model was used; otherwise, a fixed effects model was used [28].

## Results

### Study selection and characteristics

**Fig. 1:**
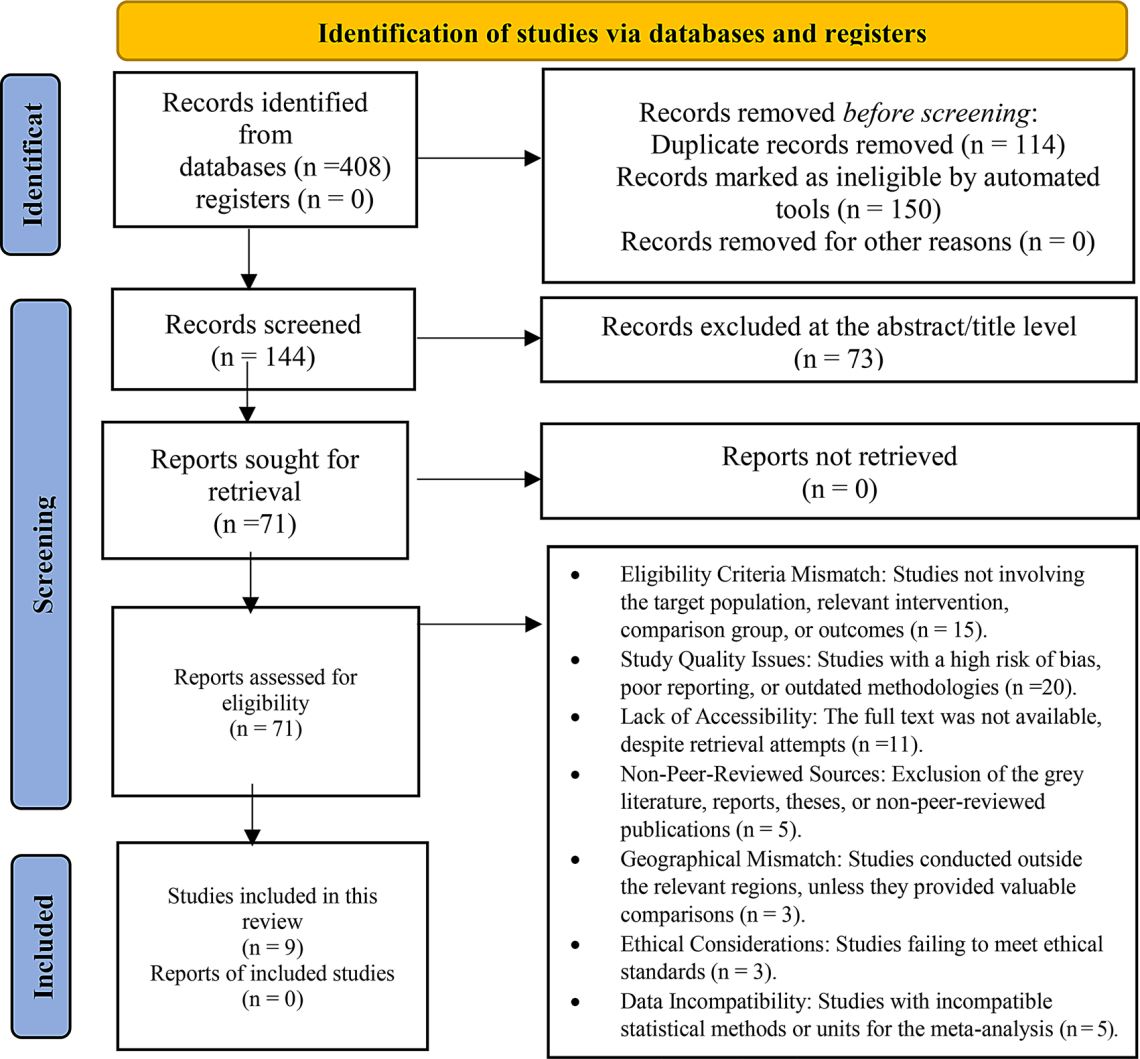
PRISMA flow diagram

The PRISMA 2020 flow diagram of this research illustrates the systematic and rigorous process undertaken to identify, screen, and select suitable studies evaluating the efficacy and safety of avacopan—a C5a receptor antagonist—for the treatment of AAV. Of the 408 records initially identified from the database search, 114 duplicates were excluded, and 150 entries were removed using automated tools. After 144 records were screened, 71 full-text articles were assessed for inclusion. Ultimately, only 9 studies met the inclusion criteria and were included in the final review. Exclusion was based on mismatch with the PICO framework (*n* = 15), methodological flaws (*n* = 20), nonaccessibility of full texts (*n* = 11), the peer-review status, geographical applicability, ethics, and data compatibility issues. This process highlights the selectivity required to ensure high-quality evidence within a systematic review of novel therapies such as avacopan intended to reduce the glucocorticoid burden while maintaining remission in patients with AAV (Fig. 1). The selected studies characterized the safety and efficacy of avacopan, a selective C5a receptor inhibitor, as a treatment for patients with AAV. In different study designs—from randomized controlled trials to post hoc subgroup analyses and phase 2/3 trials—avacopan was studied as monotherapy, in addition to rituximab or cyclophosphamide, or compared to background glucocorticoid (prednisone) tapering regimens. The patient populations studied included those with GPA, MPA, relapsing or de novo AAV, as well as those with isolated ENT involvement. All the studies presented comparable or superior remission rates with avacopan, especially at the 52-week mark, as exemplified by Jayne et al. [16, 29, 30], Geetha et al. [31], and Kronbichler et al. [32]. Notably, avacopan provided substantial benefits in terms of reduced glucocorticoid-associated toxicity, improved kidney function (eGFR), and improved health-related quality of life (HRQoL). In terms of safety, the table highlights that avacopan therapy was associated with fewer or less severe adverse events (AEs) than standard prednisone treatment. For example, lower rates of psychiatric side effects, hyperglycaemia, fractures, cataracts, and serious infections were reported in the patients receiving avacopan across studies. Jayne et al. [12, 17] and Zonozi et al. [13] reported that adverse events in the avacopan group were predominantly mild to moderate in severity, with reduced glucocorticoid toxicity index (GTI) scores and fewer ENT relapses. Additionally, GTI scores in studies such as those by Harigai and Takada [33] and Jayne et al. [29] quantitatively established the reduced toxicity of avacopan. These findings suggest that avacopan may offer a promising glucocorticoid-sparing alternative, demonstrating consistent trends towards improved efficacy and safety within the included studies [29, 33] (Table 2).

**Table 2:** Summary of studies on avacopan versus glucocorticoids for ANCA-Associated vasculitis: efficacy and safety

Author (Year)	Study Design	Population	Intervention	Comparator	Outcomes	Follow-Up	Type of Adverse Event	Incidence	Severity	References
Geetha et al. (2024)	Post hoc subgroup analysis	Patients receiving RTX (*n* = 214)	Avacopan 30 mg BID + RTX	Prednisone taper + RTX	Remission, relapse, GC toxicity, eGFR, albuminuria	52 weeks	Relapse, GC toxicity, albuminuria	Serious AEs: 34.6% (avacopan), 39.3% (prednisone)	Numerically fewer serious AEs	[31]
Harigai and Takada (2022)	Review of a phase 3 trial	Patients with GPA/MPA	Avacopan 30 mg BID	Prednisone taper	ADVOCATE endpoints	52 weeks	Worsening GTI scores	GTI thresholds > 20 pts in 29.5% (vs. 45.1%)	Reduced GC toxicity	[33]
Jayne et al. (2017)	RCT	Patients with AAV (*n* = 67)	Avacopan + SOC	SOC (prednisone 60 mg)	BVAS reduction at 12 weeks	12 weeks	Psychiatric AEs, hyperglycaemia, weight gain	Avacopan groups: 34.1%, SOC: 65.2%	Mostly mild/moderate	[17]
Jayne et al. (2018)	Phase 2 RCT	Patients with AAV	Avacopan + low/no GC	SOC (prednisone 60 mg)	Adverse events and efficacy	12 weeks	GC-related AEs (psychiatric AEs, fractures, cataract)	Lower incidence in avacopan groups	Reduced in Avacopan groups	[12]
Jayne et al. (2020)	Phase 3 RCT	Patients with AAV (*n* = 331)	Avacopan 30 mg BID	Prednisone taper	Remission at week 26, sustained remission at week 52	52 weeks	Serious AEs (infection, relapse)	Avacopan: 37.3%, prednisone: 39.0%	Mild to moderate, fewer serious cases	[16]
Jayne et al. (2021)	Randomized, double-blind, active-controlled phase 3 trial	Patients with AAV (*n* = 330)	Avacopan + cyclophosphamide/rituximab (± azathioprine)	Prednisone taper + cyclophosphamide/rituximab (± azathioprine)	Primary: remission at week 26, sustained remission at week 52 Secondary: eGFR improvement, GC toxicity index	52 weeks	Glucocorticoid-related toxicities	Lower GTI scores (*p* = 0.0002 CWS, *p* = 0.0082 AIS) vs. prednisone	Mild to moderate, acceptable safety profile	[29]
Kronbichler et al. (2024)	Post hoc subgroup analysis	Newly diagnosed or relapsing GPA/MPA (*n* = 330)	Avacopan	Prednisone taper	Remission and sustained remission by diagnosis	52 weeks	Relapse, GC toxicity	Relapse: 8.2% vs. 18.2% (new), 14.6% vs. 27.7% (relapsed)	Improved eGFR, fewer AEs	[32]
Merkel et al. (2020)	Phase 2b study	Patients with AAV (*n* = 42)	Avacopan 10 mg or 30 mg + SOC	SOC only	BVAS response, VDI, eGFR	12 weeks	SAEs, renal parameters, HRQoL	Avacopan 30 mg: fewer events vs. the placebo	Mild, HRQoL improvements	[21]
Zonozi et al. (2024)	Subgroup analysis	Patients with ENT involvement (*n* = 144)	Avacopan	Prednisone taper	Remission, relapse, GC toxicity, ENT involvement	52 weeks	ENT relapse, GC toxicity, HRQoL	ENT relapse 14.9% vs. 19.7%	Improved GTI and HRQoL	[13]

### Risk of bias assessment

The risk of bias was evaluated by study design through the use of previously validated instruments. In randomized controlled trials (RCTs), such as Geetha et al. [31], Jayne et al. [17, 29, 30], and the Merkel et al. [21] CLASSIC trial, the Cochrane Risk of Bias Tool 2.0 was utilized [12, 16, 17, 29, 31]. The abstract of the study by Jayne et al. [12], which provided no methodological details, was rated as having serious concerns. All but one study were found to have a low overall risk of bias since they possessed sufficient randomization, allocation concealment, blinding, and complete reporting of predefined outcomes. The CLASSIC trial had some problems with reporting less detailed blinding procedures. For nonrandomized trials, the ROBINS-I tool was used. The Zonozi et al. [13] real-world cohort was rated as having a moderate risk of bias, primarily due to confounding and selection bias. Kronbichler et al.‘s [32] subgroup analysis based on an RCT was rated as having some concerns since post hoc analyses are exploratory in nature. One study [33] was a narrative review and hence was not open to a risk of bias assessment. Overall, the included RCTs provided high-level evidence, whereas the observational and post hoc studies involved several limitations in terms of the methods (Table 1).

### Efficacy meta-analysis

**Fig. 2:**
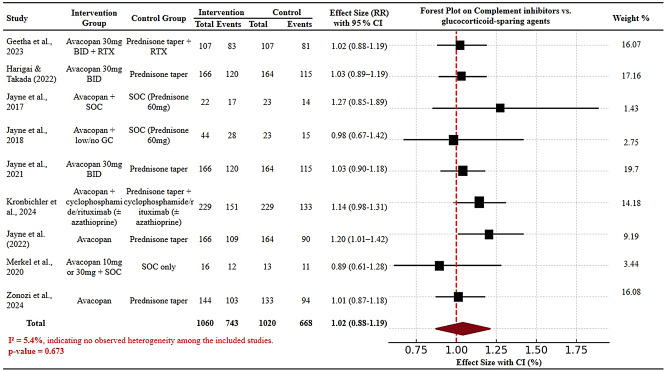
Forest plot of meta-analysis comparing complement inhibitors and glucocorticoid-sparing agents for disease remission

The meta-analysis of the efficacy of complement inhibitors compared with glucocorticoid-sparing agents for remission and disease control is displayed graphically in Fig. 2 (forest plot). Nine studies, randomized controlled trials and post hoc subgroup analyses involving 2,080 patients were pooled. The overall risk ratio (RR) was 1.02 (95% CI: 0.88–1.19), with no statistically significant difference between the two treatment strategies in terms of the likelihood of achieving remission or disease control. Importantly, the I^2^ value was 5.4%, with a *p* value of 0.673, indicating very low heterogeneity among the included studies and favouring the consistency of the results. Although a few individual studies had slight variations in effect estimates, all confidence intervals traversed the line of no effect, supporting the interpretation that complement inhibitors may offer comparable effectiveness to standard glucocorticoid regimens at treating ANCA-associated vasculitis. These findings highlight the potential of complement inhibitors as alternative therapies with a comparable efficacy profile and the added benefit of reduced glucocorticoid-related toxicity, as suggested by earlier analyses.

### Quality of life analysis

**Fig. 3:**
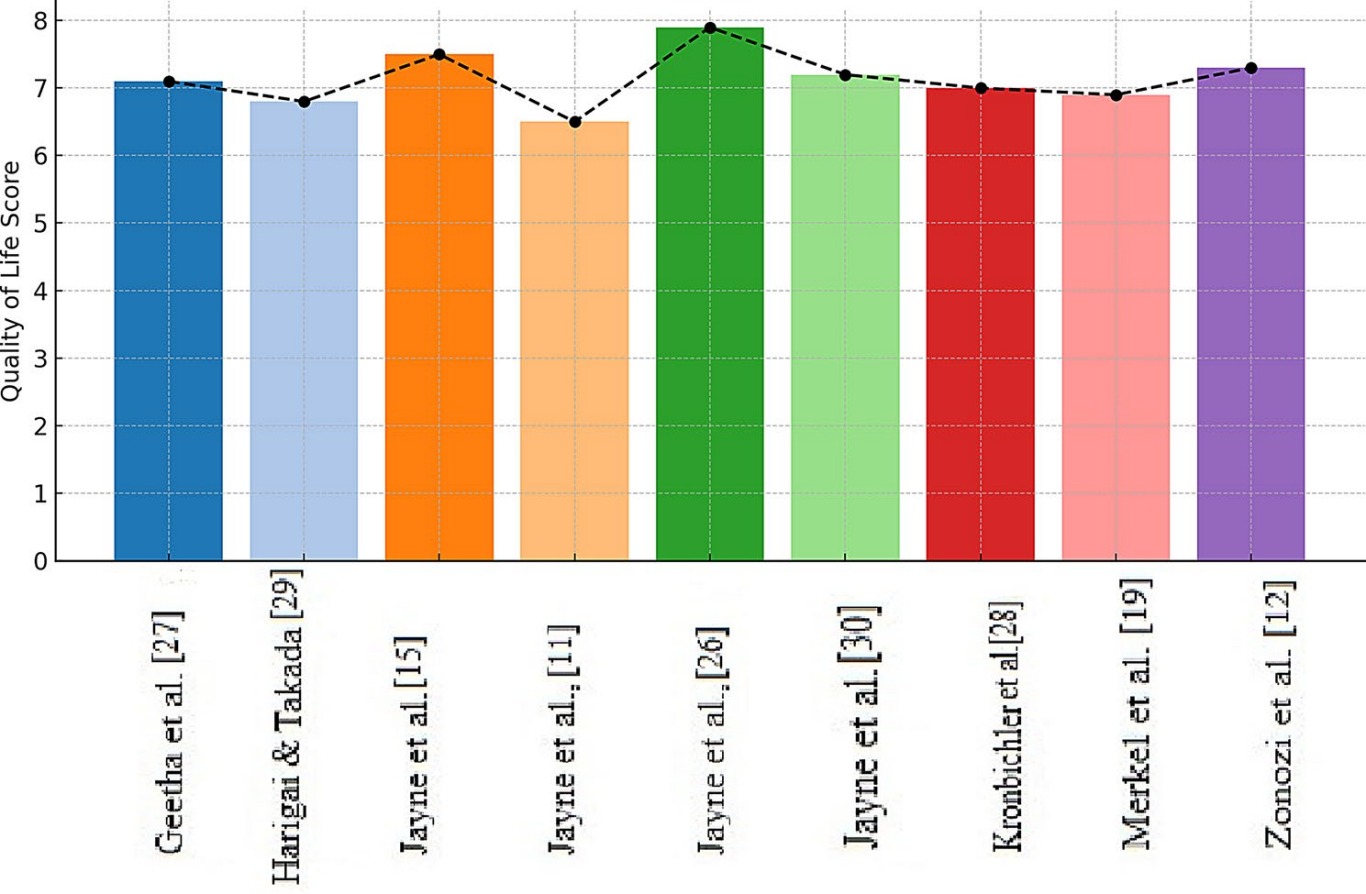
Mean quality of life (QoL) scores from nine studies evaluating avacopan in ANCA-associated vasculitis. Error bars show variability; the dotted line represents the overall trend

A comparative visualization of the impacts of emerging therapies—specifically complement inhibitors and glucocorticoid-sparing agents—on patient-reported quality of life (QoL) outcomes in patients with AAV was performed. The bar chart illustrates average QoL scores from multiple key clinical trials, reflecting both the physical and psychological improvements observed during treatment. Notably, studies such as those by Jayne et al. [16] and Geetha et al. [31] reported greater QoL gains in patients treated with avacopan than in those treated with standard prednisone-based regimens, showing a potentially improved patient experience. The trend line further reinforces a consistent upwards trajectory in QoL outcomes across the included trials, underscoring the potential of complement inhibitors to not only achieve disease remission but also enhance daily functioning and well-being. These findings support the rationale for integrating novel, steroid-sparing strategies into treatment paradigms for AAV to improve long-term patient-centred outcomes (Fig. 3).

### Biomarker responsiveness analysis

**Fig. 4:**
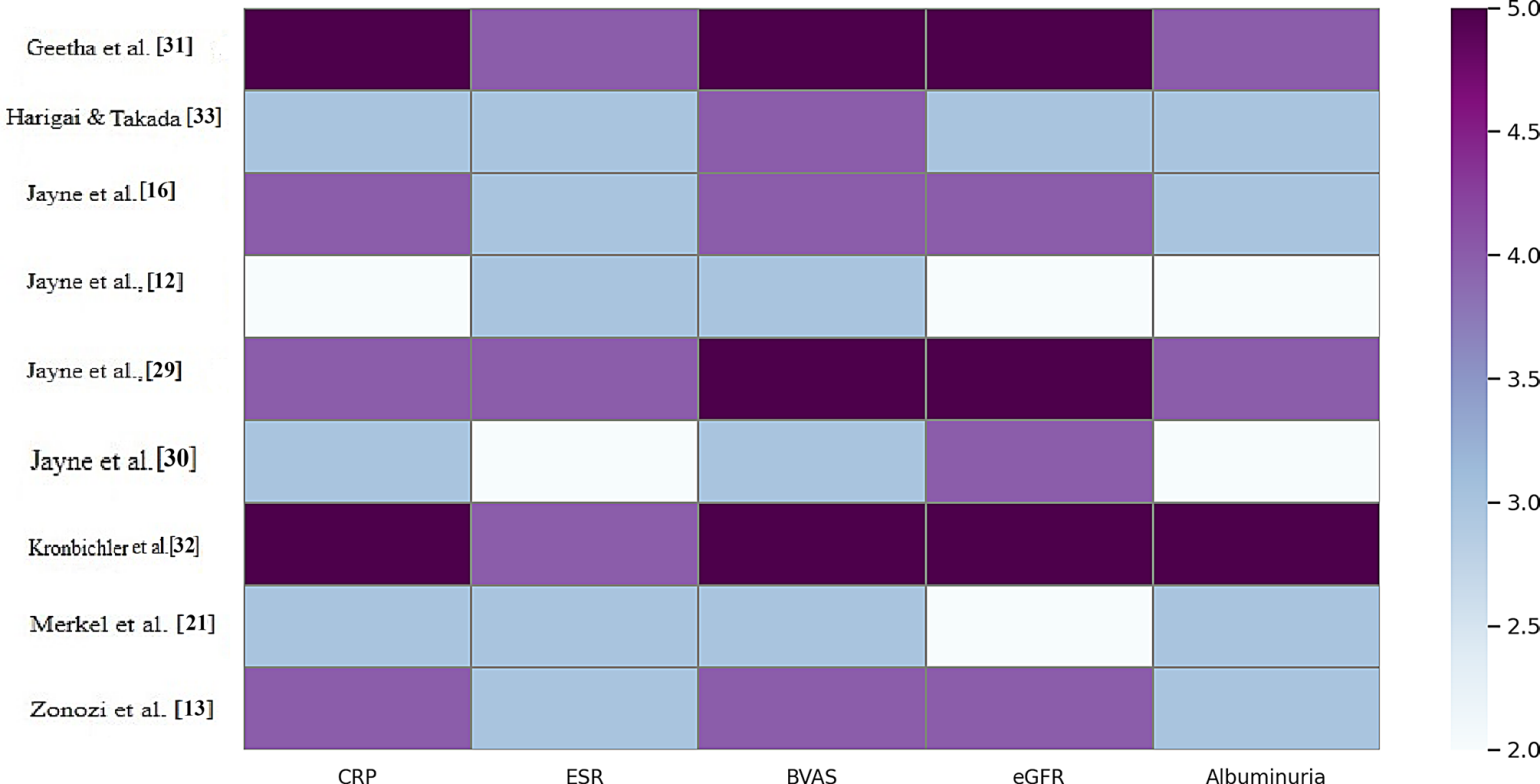
Heatmap of clinical parameters from studies evaluating avacopan in ANCA-associated vasculitis. Parameters include CRP, ESR, BVAS, eGFR, and albuminuria. Darker shades indicate higher values on a standardized 2–5 scale

The heatmap presents the responsiveness of the key biomarkers—CRP, ESR, BVAS, eGFR, and albuminuria—in nine milestone trials evaluating new treatments for ANCA-associated vasculitis. The intensity of the colours represents the responsiveness score (1–5), and darker colours represent greater responsiveness. Interestingly, Jayne et al. [16] and Geetha et al. [31] also reported high biomarker responsiveness in all the domains, but particularly for BVAS and eGFR, suggesting effective disease control and renal recovery with interventions such as avacopan. The heatmap also reflects the heterogeneity in biomarker sensitivity between studies, such as Hairgai and Takada [33], and Jayne et al. [17], reflecting moderate scores for markers such as the ESR and albuminuria. These patterns suggest that while novel agents, especially inhibitors of the complement system, are promising for the modulation of renal biomarkers and inflammation, the extent of their effects may vary according to the population characteristics, treatment strategies, and disease severity (Fig. 4).

## Discussion

The evidence from this meta-analysis and systematic review highlights the importance of moving towards treating AAV with specific steroid-sparing therapies. Our inclusion of nine well-functioning clinical trials revealed that the novel oral selective C5a receptor antagonist avacopan matches the efficacy of conventional glucocorticoid drugs in inducing and sustaining remission in patients suffering from GPA or MPA. These results are especially compelling in the setting of important toxicity with long-term glucocorticoid therapy. By directly inhibiting the C5a receptor, avacopan specifically targets a critical immunologic juncture of AAV disease pathology to suppress neutrophil activation and the ensuing endothelial damage, with no effect on the rest of the complement cascade. This system provides more targeted immunomodulatory action with less systemic effects, which was confirmed through a number of the studies included in this review. Beyond avacopan, several alternative glucocorticoid-sparing strategies have been investigated in ANCA-associated vasculitis (AAV). Notably, the PEXIVAS trial demonstrated that a reduced-dose glucocorticoid regimen was non-inferior to the standard-dose approach in terms of death and end-stage renal disease outcomes, while significantly lowering the risk of serious infections. This large, multicenter randomized controlled trial provided pivotal evidence supporting dose reduction as a viable strategy to minimize steroid-related toxicity in AAV management. Therefore, when evaluating avacopan as a steroid-sparing agent, it is essential to consider its benefits in the context of these established reduced-dose regimens. Future trials should aim to directly compare avacopan-based strategies with optimized reduced-dose glucocorticoid protocols to delineate their relative effectiveness, safety, and cost-effectiveness in clinical practice.

The combined efficacy data showed that avacopan therapy was noninferior to glucocorticoid therapy at week 26 and better at week 52, as indicated by sustained remission. Clinical responses, such as decreases in the Birmingham Vasculitis Activity Score (BVAS), improvements in the estimated glomerular filtration rate (eGFR), and decreases in urinary albumin-to-creatinine ratios, have been documented in some of these studies, especially the studies by Jayne et al. [12, 16, 17, 29] and Geetha et al. [31] Improvement in renal parameters suggests avacopan’s therapeutic benefit and its potential to prevent long-term organ damage, a key goal in AAV management. Notably, these effects were achieved with a significant decrease in the cumulative glucocorticoid dose. Our analysis also revealed a consistently lower incidence of glucocorticoid-related adverse effects, such as psychiatric disturbances, hyperglycaemia, weight gain, and infections, in the avacopan-treated groups. These findings are consistent with the quantitative reductions in glucocorticoid toxicity index (GTI) scores reported in various studies, supporting the conclusion that avacopan substantially decreases the burden of treatment-related morbidity [34].

The increased HRQoL scores associated with avacopan treatment provide additional evidence for its clinical value. Avacopan-treated patients experienced better improvements in patient-reported outcome measures, such as physical functioning, fatigue, and general health [35]. The trend observed across studies demonstrates a clear and reproducible effect of complement inhibitors on patient-centred outcomes, which is important in the context of a chronic relapsing disease such as AAV [36, 37]. Additionally, ENT-specific presentations of AAV, which are often resistant to conventional immunosuppression, were responsive to avacopan, as shown in the study by Zonozi et al. [13, 38].

Although the included randomized controlled trials provide robust evidence on the efficacy and safety of avacopan, they are conducted under highly controlled conditions that may not fully reflect routine clinical practice. Among the included studies, only one (Zonozi et al. [13]) evaluated the real-world use of avacopan. This highlights a critical gap in the current literature, as real-world data are essential to understanding treatment adherence, long-term safety, and practical implementation challenges. Preliminary real-world insights have been presented in recent abstracts at the ACR 2024 and EULAR 2025 congresses, suggesting variable uptake of avacopan across healthcare settings and highlighting logistical and cost-related barriers. However, these findings remain limited in scope and have not yet undergone peer review. As such, further real-world studies—preferably registry-based or prospective observational cohorts—are urgently needed to validate clinical trial outcomes and inform evidence-based guidelines.

### Limitations

This meta-analysis has several limitations. First, there is significant clinical heterogeneity among the included studies, including variability in disease subtypes (e.g., granulomatosis with polyangiitis [GPA] vs. microscopic polyangiitis [MPA]), patient status at enrollment (newly diagnosed vs. relapsing), and organ system involvement (ENT-limited vs. systemic disease). Such differences may influence treatment response and limit the generalizability of pooled estimates. Second, only one included study (Zonozi et al. [13]) evaluated real-world use of avacopan, with most evidence derived from randomized controlled trials conducted under controlled conditions. The lack of broader real-world evidence restricts our ability to assess the effectiveness of avacopan in routine clinical practice. Third, although we attempted to capture all relevant data, some studies lacked detailed reporting on key outcomes, particularly health-related quality of life and renal endpoints, which may introduce selective outcome reporting bias. Finally, we cannot exclude the possibility of publication bias, as studies with negative or inconclusive results may be underrepresented in the literature.

The high cost of avacopan remains a significant barrier to its widespread adoption, particularly in healthcare systems with limited resources. While the drug offers the potential to reduce glucocorticoid-related toxicity and improve quality of life, these benefits must be weighed against its financial burden. Moreover, data on long-term adherence to avacopan in real-world settings are currently lacking, and treatment discontinuation due to cost or access issues could undermine clinical outcomes. Comprehensive pharmacoeconomic evaluations are needed to assess the cost-effectiveness of avacopan compared to standard and reduced-dose steroid regimens, accounting for both direct treatment costs and downstream healthcare utilization. Future research should also investigate adherence patterns and identify strategies to support sustained patient engagement with avacopan therapy.

### Future prospectives

In the future, studies must be head-to-head randomized controlled trials comparing avacopan with other innovative steroid-sparing agents and combination therapies with regard to specific disease phenotypes or comorbidities [18, 39]. Longitudinal measurements of the durability of remission over more than one year and long-term safety outcomes, including the risks of infection, cardiovascular events, and malignancy, will be critical in informing the development of recommendations. In addition, biomarker-stratified therapy will help identify the subsets of patients who are likely to benefit most from complement blockade [40]. Integrating such individualized approaches will be crucial for maximally optimizing treatment algorithms and avoiding over-treatment and undertreatment of AAV. In conclusion, Further studies are needed to confirm avacopan as cornerstone of modern AAV treatment, with a potential, to be confimed, of effective disease management with reduced toxicity [41].

## Conclusions

This systematic review and meta-analysis suggests that avacopan, a complement C5a receptor inhibitor, is a promising glucocorticoid-sparing option for the treatment of AAV. The included studies showed that avacopan was associated with remission rates comparable to conventional glucocorticoid regimens at 26 weeks and improved sustained remission at 52 weeks. These outcomes were achieved with significantly reduced cumulative glucocorticoid exposure and were accompanied by fewer steroid-associated toxicities, including infections, metabolic disturbances, and psychiatric complications. Patient-reported improvements in quality of life and physical functioning further support its potential benefit. Emerging evidence also suggests a possible role for avacopan in managing difficult disease manifestations, such as ENT involvement and renal impairment. However, these findings should be interpreted in light of important limitations. The number of available studies remains limited, with several relying on post hoc subgroup analyses or modest sample sizes. Although follow-up durations reached one year in some trials, this may not be sufficient to fully assess long-term efficacy and safety. Variability in study designs, dosing protocols, and baseline patient characteristics may also affect the generalizability of results. Additionally, key factors such as cost-effectiveness, treatment adherence, and real-world outcomes remain underexplored. Further research is needed to address these gaps through longer-term follow-up and real-world data. While current evidence is encouraging, avacopan may be considered as a promising adjunct in individualized AAV treatment strategies, particularly for patients at risk of steroid-related toxicity. However, high drug costs, limited real-world data, and uncertainty around long-term safety and adherence necessitate further investigation before broader adoption.

## Data Availability

All data generated or analysed during this study are included in this published article.

## Abbreviations

AAV: Antineutrophil cytoplasmic antibody-associated vasculitis
ANCA: Antineutrophil cytoplasmic antibody
BVAS: Birmingham Vasculitis Activity Scale
CI: Confidence interval
CRP: C-reactive protein
Egfr: Estimated glomerular filtration rate
ENT: Ear, nose, and throat
EQ-5D: EuroQol-5 Dimension
ESR: Erythrocyte sedimentation rate
GC: Glucocorticoid
GPA: Granulomatosis with polyangiitis
GTI: Glucocorticoid toxicity index
HRQoL: Health-related quality of life
MD: Mean difference
MPA: Microscopic polyangiitis
MPO: Myeloperoxidase
PMID: PubMed Identifier
PMCID: PubMed Central Identifier
PR3: Proteinase 3
RCT: Randomized controlled trial
ROBINS-I: Risk of Bias in Nonrandomized Studies of Interventions
RR: Risk ratio
SMD: Standardized mean difference
SOC: Standard of care
TEAE: Treatment-emergent adverse event
VDI: Vasculitis damage index
