# Correction: Efficacy and safety of avacopan in the treatment of ANCA-associated vasculitis: a systematic review and meta-analysis

**DOI:** 10.1186/s41927-025-00609-5

**Published:** 2025-12-24

**Authors:** Khaled Aldhuaina, Khawla Alghanim

**Affiliations:** Rheumatology Unit, Department of Internal Medicine, King Fahad Military Medical Complex, Dhahran, Saudi Arabia

**Correction: BMC Rheumatol 9, 115 (2025).**

10.1186/s41927-025-00569-w

Following publication of the original article [1], the author found that in Fig. 2, the column heading “Effect Size (RR) with 9%% CI” should correctly read “Effect Size (RR) with 95% CI.”

Therefore, Fig. 2 has been corrected from:

**Figure:**
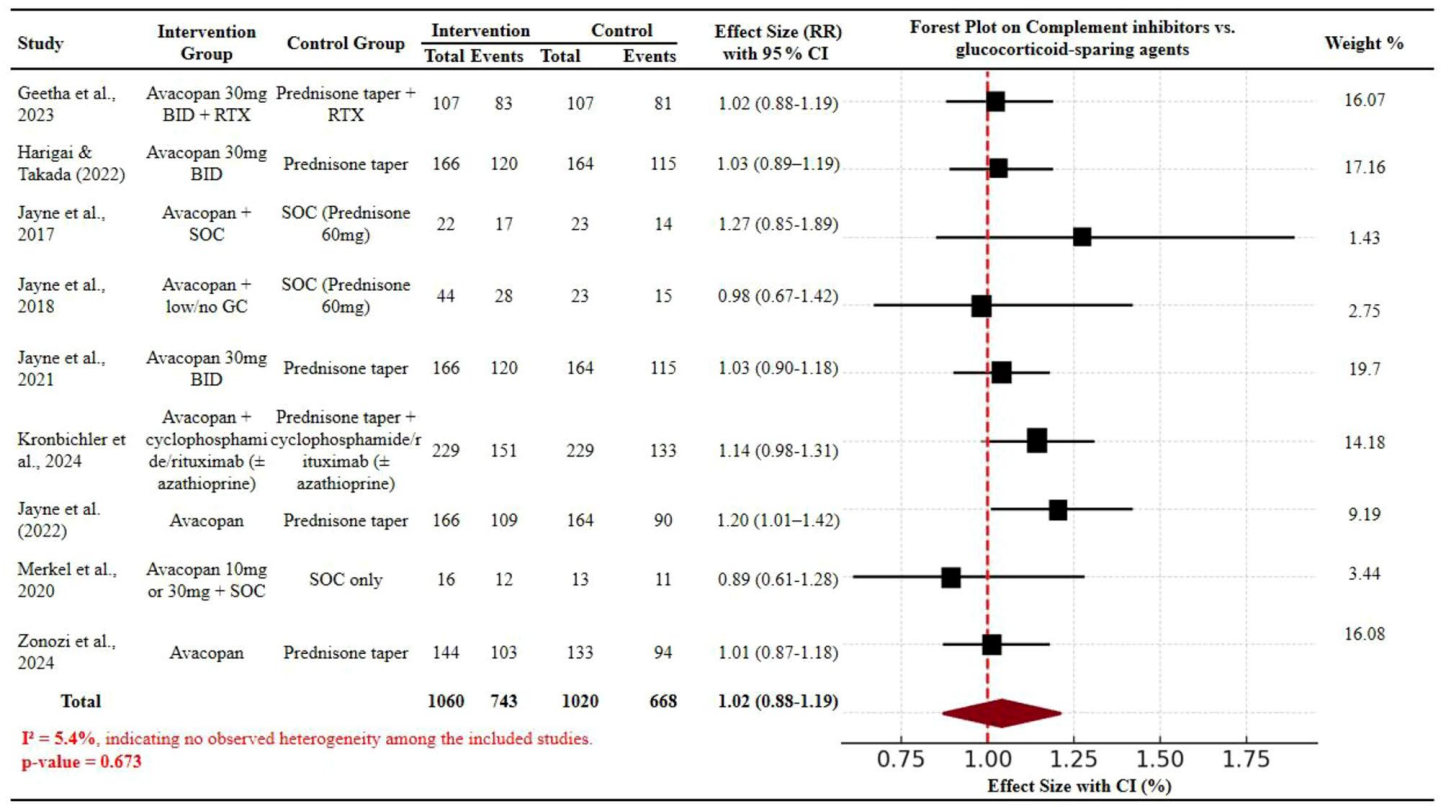

To:

**Fig. 2:**
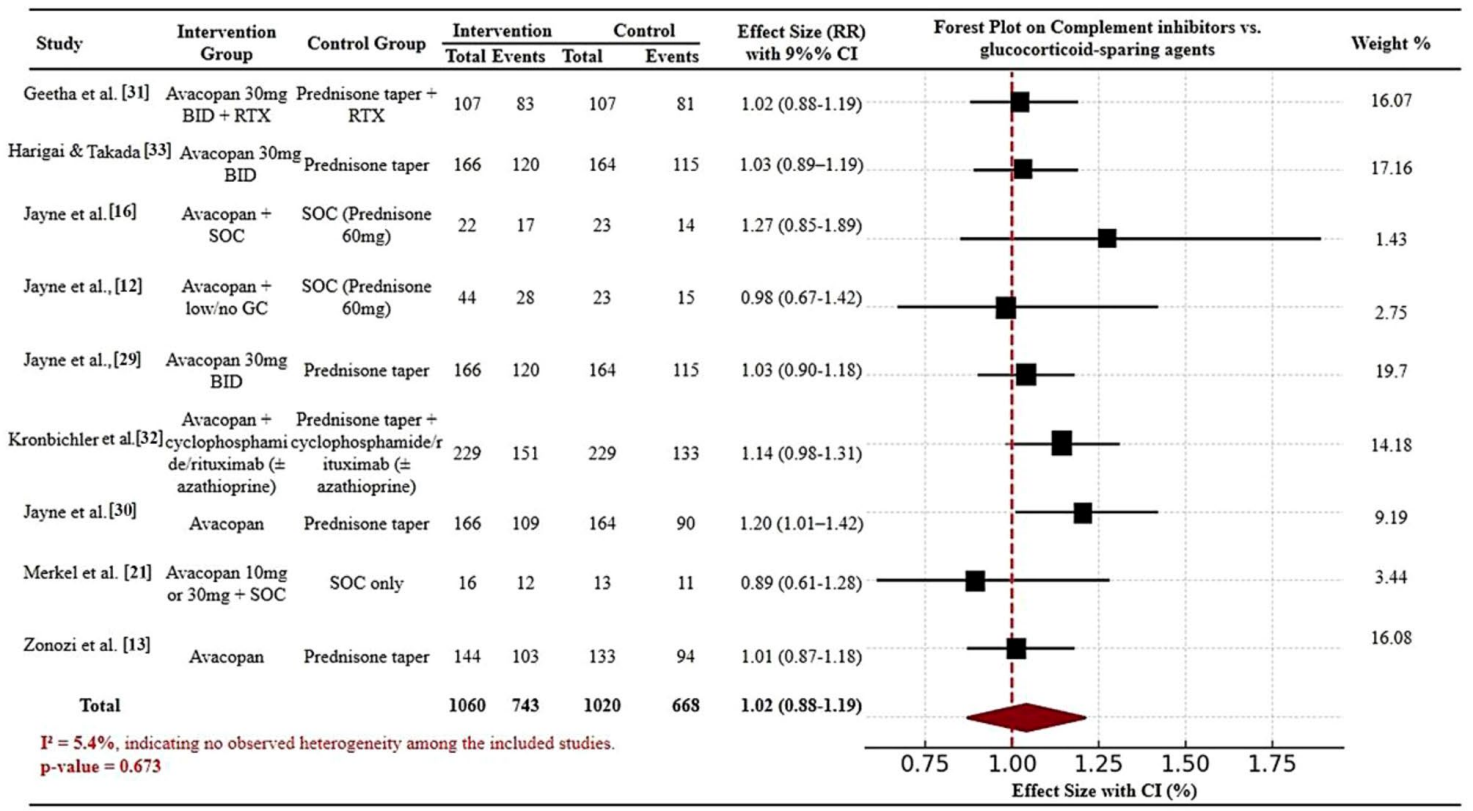
Forest plot of meta-analysis comparing complement inhibitors and glucocorticoid-sparing agents for disease remission

The original article [1] has been updated.
